# Machine learning algorithms for predicting glycemic control and weight loss outcomes in GLP-1 receptor agonist users

**DOI:** 10.3389/frai.2026.1861563

**Published:** 2026-07-15

**Authors:** Tadesse M. Abegaz, Gabriel Frietze

**Affiliations:** School of Pharmacy, University of Texas at El Paso, El Paso, TX, United States

**Keywords:** All of US research program, explainable artificial intelligence, GLP-1 receptor agonists, glycemic control, machine learning, weight loss

## Abstract

**Introduction:**

Glucagon-like peptide-1 receptor agonists (GLP-1 RAs) are widely used for the management of type 2 diabetes mellitus and obesity; however, substantial inter-individual variability in glycemic and weight loss outcomes remains. This study aimed to develop and validate machine learning (ML) models to predict glycemic control and weight loss outcomes following GLP-1 RA initiation using real-world data and to identify key features associated with treatment response.

**Methods:**

We conducted a retrospective cohort study using data from the All of Us Research Program. Adult participants initiating GLP-1 RA therapy with available baseline and follow-up measurements were included. Two cohorts were constructed: a glycemic control cohort (*n* = 3,975) and a weight loss outcome cohort (*n* = 11,420). Glycemic control was defined as hemoglobin A1c (HbA1c) <7% at follow-up, and weight improvement was defined as achieving a body mass index (BMI) <30 kg/m². Multiple ML models, including logistic regression, random forest (RF), extreme gradient boosting (XGBoost), support vector machine, neural network, LightGBM, and CatBoost, were developed using 10-fold cross-validation. Model performance was evaluated using area under the receiver operating characteristic curve (AUC), accuracy, precision, recall, and precision–recall curves. SHapley Additive exPlanations (SHAP) were used to improve model interpretability.

**Results:**

For weight loss outcome prediction, ensemble models demonstrated superior performance, with RF and XGBoost achieving the highest discrimination (AUC ≈ 0.94) and accuracy (0.89–0.90). For glycemic control prediction, RF and XGBoost achieved modest performance (accuracy ≈ 0.73; AUC ≈ 0.79). SHAP analysis identified baseline BMI and body weight as the most influential features of weight improvement, while duration of diabetes, baseline HbA1c, and use of sulfonylureas or insulin were among the most important features of glycemic control.

**Discussion:**

Machine learning models, particularly tree-based ensemble methods, demonstrated strong potential for predicting treatment response to GLP-1 RA therapy. Integration of explainable ML approaches with real-world data may support personalized treatment strategies, and facilitate identification of patients most likely to benefit from GLP-1 RA therapy.

## 1 Introduction

Glucagon-like peptide-1 receptor agonists (GLP-1 RAs) have emerged as a cornerstone therapy for the management of type 2 diabetes mellitus (T2DM) and obesity, offering clinically meaningful improvements in glycemic control alongside significant weight reduction (Zheng et al., 2024; Rao, 2026). Despite their well-established efficacy, substantial inter-individual variability in treatment response persists, with a considerable proportion of patients failing to achieve optimal glycemic or weight loss outcomes (Squire et al., 2025). This heterogeneity in response presents a critical challenge for clinicians and underscores the need to identify responders and patient characteristics associated with treatment response which helps to personalize GLP-1 RA therapy (Dawed et al., 2023; Norbury and Seymour, 2018).

Traditional approaches to predicting health outcomes have been employed using individual clinical characteristics; however, these methods often fail to capture complex, non-linear relationships among patient-level factors (Connery et al., 2020). Machine learning (ML) techniques, on the other hand, provide a powerful alternative by enabling the integration and analysis of high-dimensional clinical data to identify patterns that may not be apparent using conventional statistical methods (Rajula et al., 2020). In recent years, ML has demonstrated considerable potential in improving risk prediction, treatment optimization, and clinical decision-making across a range of healthcare domains screening (Norbury and Seymour, 2018; Alowais et al., 2023). Nevertheless, its application in predicting glycemic control and weight loss outcomes among patients receiving GLP-1 RA therapy remains limited.

The increasing adoption of GLP-1 RAs in routine clinical practice, coupled with the growing availability of large-scale real-world data, presents a unique opportunity to develop predictive models that can inform individualized treatment strategies. Leveraging rich clinical datasets, ML models can be used to identify key features associated with treatment response and stratify patients according to their likelihood of achieving clinically meaningful outcomes (Abualigah et al., 2025). Early prediction of treatment responses have the potential to enhance treatment selection, improve patient outcomes, and reduce unnecessary healthcare costs associated with ineffective therapies (Khalifa and Albadawy, 2024).

In this study, we applied multiple supervised machine learning algorithms to predict glycemic control and weight loss outcomes among individuals receiving GLP-1 RA therapy using real-world clinical data from the All of Us (AoU) Research Program (Ramirez et al., 2022). Specifically, we aimed to Zheng et al. (2024) develop and validate predictive models for glycemic control and weight loss outcome and (Rao, 2026) identify key patient characteristics associated with treatment response. This study has several important novel contributions. First, it leverages the large and diverse AoU dataset, which includes substantial representation of populations historically underrepresented in biomedical research. Second, unlike many previous studies focusing on a single endpoint, this study simultaneously evaluates prediction of both glycemic and weight loss outcomes following GLP-1 RA initiation (Thomas et al., 2015). In addition, the study integrates explainable artificial intelligence techniques to improve model interpretability and clinical relevance.

## 2 Materials and methods

### 2.1 Study design and data source

We conducted a retrospective cohort study using de-identified data from the AoU Research Program, a large, nationwide United State (U.S) data that integrates electronic health records (EHRs), survey responses, physical measurements, medical condition, medication history and genomic data (AoURP, 2019). The AoU program includes participants from diverse demographic and clinical backgrounds, with substantial representation of populations historically underrepresented in biomedical research. As of February 2025, the AoU Research Program constitutes 633,547 participants, of which 77% of participants recruited from communities historically underrepresented in biomedical research, and 46% self-identify as members of underrepresented racial and ethnic minority groups (Ramirez et al., 2022; Marees et al., 2018; The All of Us Research Program Genomics Investigators, 2024).

### 2.2 Study population

The study included adult participants with evidence of GLP-1 RA use and sufficient clinical data to evaluate treatment response. Two analytic cohorts were constructed: a glycemic control cohort and a weight loss outcome cohort. For the glycemic control cohort, participants were required to have a baseline HbA1c measurement prior to GLP-1 RA initiation and a follow-up HbA1c measurement 180 days after initiation. For the weight outcome cohort, participants were required to have baseline and follow-up measures of body weight and/or body mass index (BMI) within the same time window. Participants with missing outcome data or implausible measurements were excluded from the corresponding analysis. Cohorts were analyzed separately because availability of laboratory and anthropometric measurements differed across participants.

**Figure 1:**
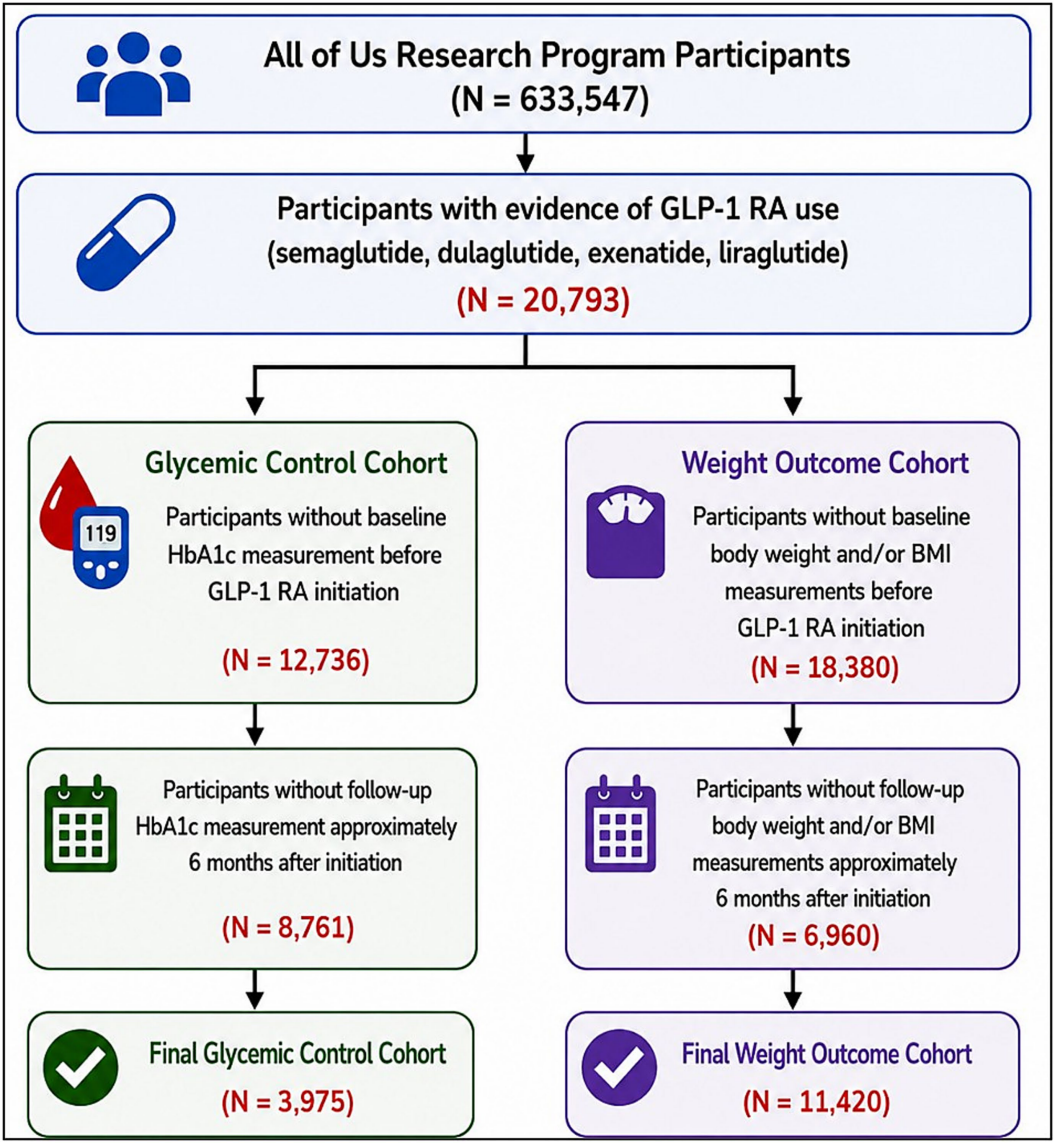
Participant selection and cohort construction for prediction of weight loss and glycemic control following GLP-1 RA therapy.Flow diagram illustrating the selection of participants from the All of Us Research Program for the development of machine learning models predicting glycemic control and weight loss outcomes following initiation of glucagon-like peptide-1 receptor agonist (GLP-1 RA) therapy. Of 633,547 participants in the database, 20,793 had evidence of GLP-1 RA use. After applying outcome-specific eligibility criteria requiring baseline and follow-up clinical measurements, 3,975 participants were included in the glycemic control cohort, and 11,420 participants were included in the weight loss outcome cohort.

The flowchart in Figure 1 illustrates the sequential participant selection process used to construct the glycemic control and weight loss outcome cohorts from the AoU Research Program dataset. Among 633,547 participants enrolled in the Program, 20,793 participants had evidence of GLP-1 RA use. For the glycemic control cohort, participants without baseline HbA1c measurements prior to GLP-1 RA initiation were excluded (*n* = 12,736), followed by exclusion of participants without follow-up HbA1c measurements (*n* = 8,761). The final glycemic control cohort included 3,975 participants. For the weight loss outcome cohort, participants without baseline body weight and/or body mass index (BMI) measurements prior to GLP-1 RA initiation were excluded (*n* = 2,413), followed by exclusion of participants without follow-up body weight and/or BMI measurements (*n* = 6,960). The final weight loss outcome cohort included 11,420 participants (Figure 1).

### 2.3 Exposure definition

Exposure was defined as initiation of a GLP-1 RA identified from medication records in the AoU database. GLP-1 RA agents included semaglutide, dulaglutide, exenatide, and liraglutide. The index date was defined as the date of first recorded GLP-1 RA use. Baseline covariates were derived from data recorded before or at the time of treatment initiation.

### 2.4 Measurement of glycemic and weight loss outcomes

Clinical outcomes were assessed using standardized measures available within the AoU Research Program dataset. For the glycemic control analysis, the outcome was defined as achieving HbA1c below 7% at follow-up after GLP-1 RA initiation. This threshold was selected based on the American Diabetes Association (ADA) Standards of Medical Care in Diabetes, which recommend this as an appropriate glycemic target for many nonpregnant adults with diabetes (Amer Diabet Assoc Professional Practice Comm NA et al., 2025). In addition, this cutoff has been associated with lower rates of diabetes-related microvascular complications and is widely used as a clinically meaningful treatment target.

For the weight loss outcome analysis, weight improvement was operationalized using post-treatment obesity status, with participants classified as improved if their follow-up BMI was below30 kg/m^2^ (Franch-Nadal et al., 2022). Baseline HbA1c was defined as the closest value recorded before GLP-1 RA initiation. Follow-up HbA1c and weight-related measurements were defined as the closest available values recorded 6 months after treatment initiation (Dawed et al., 2023). In addition, we further classified the weight loss outcome in terms of clinically meaningful weight loss which is defined as a weight loss about 5% from the baseline (Swift et al., 2016).

### 2.5 Predictor variables

Candidate predictors were selected *a priori* based on clinical relevance and data availability within the dataset. These variables included demographic characteristics such as age, sex/gender, race, ethnicity, and marital status; socioeconomic factors including education, employment, insurance status, and annual income; and clinical and metabolic measures such as baseline HbA1c, baseline BMI, baseline weight, lipid measures, blood pressure, and duration of diabetes when available. We also included comorbid conditions, including diabetes, chronic kidney disease, and heart failure, as well as medication-related variables such as GLP-1 RA type and concomitant use of insulin, metformin, sulfonylureas, sodium-glucose cotransporter-2 inhibitors (SGLT2i), dipeptidyl peptidase-4 inhibitors (DPP-4i), statins, and other selected medications. To preserve temporal ordering between predictors and outcomes, only variables measured before or at the time of GLP-1 RA initiation were included in the predictive models.

### 2.6 Data processing and analysis

All analyses were conducted within the AoU Researcher Workbench using Python-based machine learning workflows. Data management, preprocessing, model development, and evaluation were implemented using reproducible analytic pipelines. Data preprocessing was performed separately for the glycemic control and weight loss outcome cohorts. Continuous variables were summarized using means and standard deviations, whereas categorical variables were summarized using frequencies and percentages. Prior to model development, continuous variables were standardized when required by specific algorithms. Categorical variables were transformed using one-hot encoding. Missing data were handled using multiple imputation by chained equations, model-compatible imputation procedure (Azur et al., 2011). To avoid information leakage, all preprocessing procedures, including imputation, scaling, encoding, and oversampling, were performed separately within the training data during cross-validation. Random oversampling was applied to the training data within each cross-validation fold to improve class representation during model development. This approach was implemented to minimize bias and avoid artificial inflation of model performance estimates.

### 2.7 Machine learning model development

We developed supervised machine learning models to predict glycemic control and weight improvement outcomes following initiation of GLP-1 RA therapy. The evaluated algorithms included logistic regression (LR), randomForest (RF), extreme gradient boosting (XGBoost), support vector machine (SVM), neural network (NN), light gradient boosting machine (LightGBM), and CatBoost. Tree-based ensemble methods were included because of their ability to capture complex nonlinear relationships and higher-order interactions among predictors, whereas logistic regression served as a benchmark traditional classification model. Neural network models were additionally evaluated to determine whether more flexible nonlinear architectures could further improve predictive accuracy (Rainio et al., 2024).

For each outcome, the dataset was randomly partitioned into training (80%) and testing (20%) subsets using stratified sampling to preserve the original class distribution. Model development and hyperparameter tuning were conducted within the training dataset, while the held-out test set was reserved for final independent evaluation.

To further assess model stability and reduce overfitting, 10-fold stratified cross-validation was implemented during model training. Hyperparameter optimization was conducted using grid-search-based cross-validation procedures within the training data. The evaluated hyperparameters included tree depth, learning rate, number of estimators, regularization parameters, and kernel parameters depending on the specific algorithm. All preprocessing steps, including imputation, encoding, scaling, and oversampling, were incorporated within the cross-validation training pipelines to prevent information leakage.

### 2.8 Model performance evaluation

Model performance was evaluated using multiple discrimination and classification metrics, including accuracy, precision, recall (sensitivity), F1-score, and area under the curve (AUC). Because class imbalance can limit the interpretability of accuracy alone, precision-recall (PR) curves and the area under the precision-recall curve (PR-AUC) were also examined. Models with higher AUC and PR-AUC, while maintaining balanced precision and recall, were considered to have superior predictive performance.

### 2.9 Model explainability and sensitivity analysis

To improve interpretability of the best-performing models, we applied SHapley Additive exPlanations (SHAP) to quantify the contribution of each feature to the predicted outcome (Lundberg and Lee, 2017). SHAP summary plots were generated separately for glycemic control and weight outcome models to identify the most influential predictors and to characterize the direction and magnitude of their effects on model predictions. In addition, we conducted a sensitivity analysis by evaluating model performance at the two separate definitions of weight loss outcomes measures and by sequential removing of features from the model.

## 3 Results

### 3.1 Characteristics of participants in the weight loss cohort

A total of 11,420 participants receiving GLP-1 RA therapy were eligible for the weight loss cohort, of whom 2,416 (21.2%) achieved weight improvement. The majority of participants were female (65.2%), with a mean age of 55.11 ± 12.53 years. The cohort was predominantly White (51.4%), and most participants were non-Hispanic (78.8%). Regarding GLP-1 RA therapy, semaglutide was the most commonly used agent (39.6%), followed by dulaglutide (27.3%) and exenatide (25.8%). At baseline, the mean hemoglobin A1c (HbA1c) was 7.20 ± 1.75%, the mean body weight was 106.21 ± 27.77 kg, and the mean body mass index (BMI) was 37.41 ± 7.86 kg/m^2^ (See Supplementary Table S1).

### 3.2 Characteristics of participants in the glycemic control cohort

A total of 3,975 participants were eligible for the glycemic control cohort, of which 2,549 (64.1%) had controlled glycemic status. The cohort consisted of 54.4% males. Most participants were White (54.9%), and the majority were non-Hispanic (80.2%). The mean age was 58.93 ± 11.76 years. Regarding GLP-1 RA therapy, dulaglutide (33.9%) and semaglutide (30.9%) were the most used agents, followed by liraglutide (25.4%). The mean BMI was 35.84 ± 9.31 kg/m^2^, and the mean baseline HbA1c was 8.35 ± 1.87%. The average duration of diabetes was 11.34 ± 5.14 years. Additional information on the characteristics of participants is available in the Supplementary Table S2.

### 3.3 ML model performance for predicting weight loss outcomes

**Figure 2:**
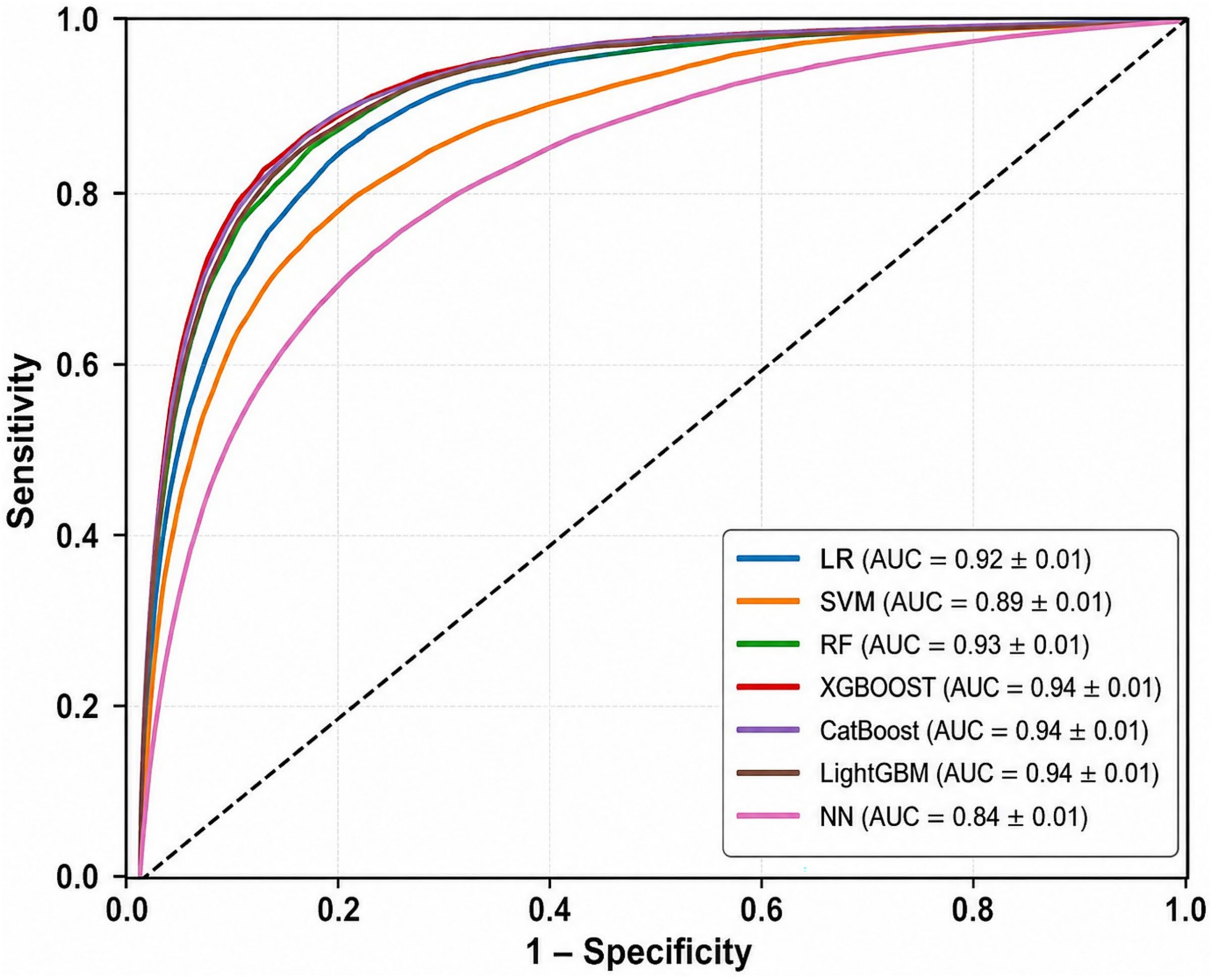
10-fold cross-validated receiver operating characteristic (ROC) curves for prediction of weight loss following GLP-1 RA therapy.Receiver operating characteristic (ROC) curves illustrating the performance of multiple machine learning models for predicting weight loss among GLP-1 RA users. The X-axis represents false positive rate while the Y-axis denotes true positive rate (sensitivity). Model performance was evaluated using 10-fold cross-validation. Ensemble models, including Random Forest, XGBoost, LightGBM, and CatBoost, demonstrated superior discrimination compared with traditional approaches. The strong predictive performance observed for these models suggests that patient demographic, clinical, laboratory, and treatment-related characteristics can be used to identify individuals more likely to achieve favorable weight outcomes following GLP-1 RA initiation.

Across all evaluated machine learning models, predictive performance varied by algorithm for weight loss prediction. Overall, tree-based ensemble methods demonstrated superior performance compared to traditional models. The RF and XGBoost models achieved the highest overall performance, with both demonstrating strong discrimination (AUC: 0.94 ± 0.01). XGBoost achieved the highest accuracy (0.90 ± 0.01), followed by RF (0.89 ± 0.01). RF demonstrated the highest precision (0.81 ± 0.03), indicating better ability to correctly identify individuals achieving clinically meaningful weight loss, whereas XGBoost showed slightly higher sensitivity (0.70 ± 0.03 vs. 0.65 ± 0.02), suggesting improved detection of true responders (Table 1 and Figure 2).

**Table 1:** Performance of machine learning model for weight loss prediction.

Model	Accuracy	Precision	Sensitivity	F1-score
LR	0.81 ± 0.01	0.53 ± 0.02	0.88 ± 0.02	0.66 ± 0.02
RF	0.89 ± 0.01	0.81 ± 0.03	0.65 ± 0.02	0.72 ± 0.02
XGBoost	0.90 ± 0.01	0.79 ± 0.03	0.70 ± 0.03	0.74 ± 0.02
SVM	0.82 ± 0.01	0.55 ± 0.02	0.78 ± 0.01	0.64 ± 0.01
NN	0.83 ± 0.01	0.61 ± 0.03	0.56 ± 0.03	0.58 ± 0.02
LightGBM	0.88 ± 0.01	0.68 ± 0.02	0.82 ± 0.03	0.74 ± 0.02
Catboost	0.88 ± 0.01	0.69 ± 0.03	0.81 ± 0.03	0.74 ± 0.02

### 3.4 Model performance for predicting glycemic control

**Figure 3:**
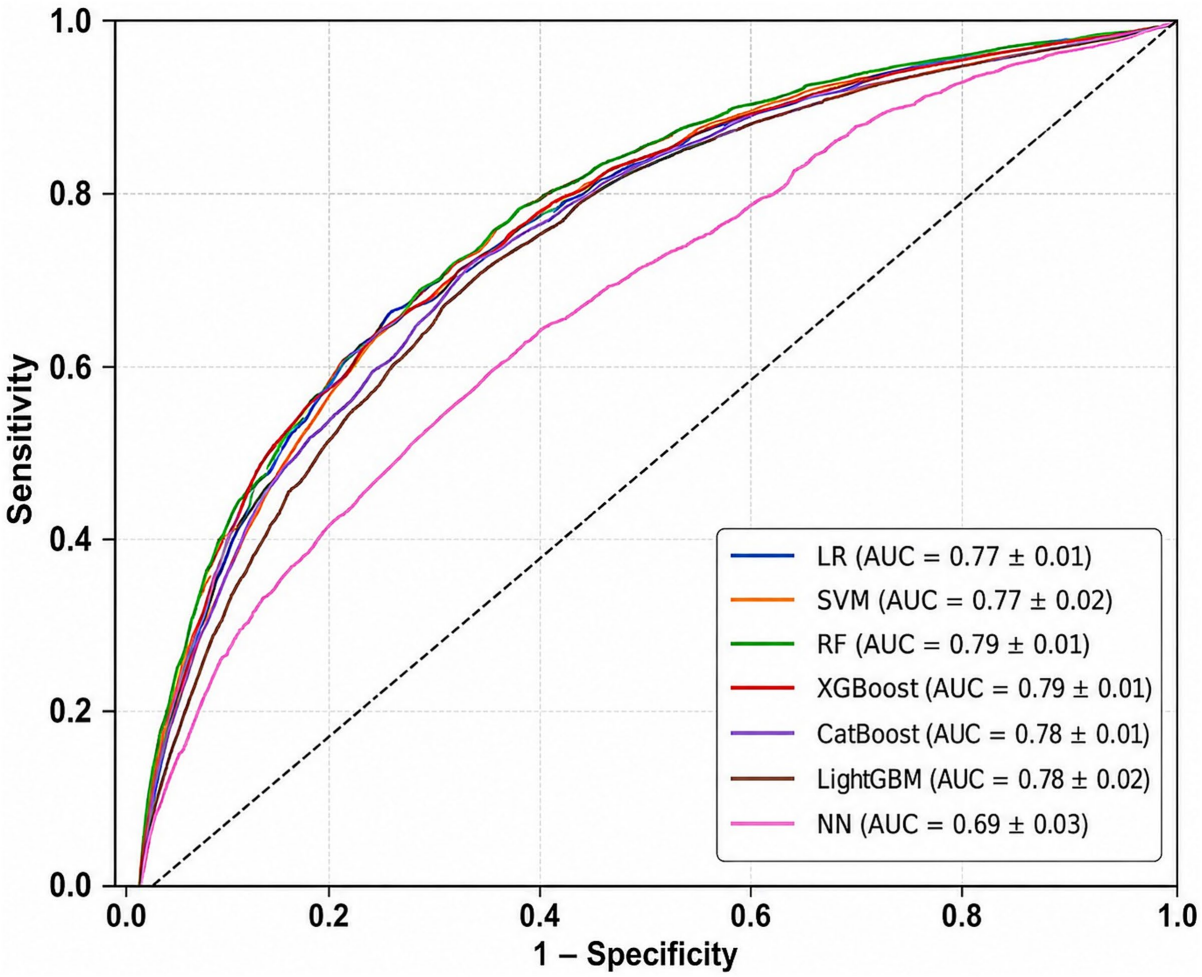
10-fold cross-validated receiver operating characteristic (ROC) curves for prediction of glycemic control following GLP-1 RA therapy.Receiver operating characteristic (ROC) curves demonstrating the performance of multiple machine learning models for predicting glycemic control among individuals receiving GLP-1 RA therapy. Model performance was evaluated using 10-fold cross-validation. The diagonal dashed line represents the line of no discrimination. Although predictive performance was more modest than for weight outcomes, tree-based ensemble models consistently achieved the best discrimination. These findings suggest that machine learning approaches may help identify patients who are more likely to achieve clinically meaningful glycemic improvement following treatment initiation. Results are based on 10-fold cross-validation.

Predictive performance for glycemic control demonstrated moderate discrimination and accuracy across all evaluated models. Among the evaluated models, RF and XGBoost demonstrated relatively strong and balanced performance (accuracy = 0.73 ± 0.01). LightGBM and CatBoost showed comparable performance, with relatively balanced precision (0.79 ± 0.01) (Table 2 and Figure 3).

**Table 2:** Performance of machine learning model for glycemic control prediction.

Model	Accuracy	Precision	Sensitivity	F1-score
LR	0.67 ± 0.01	0.66 ± 0.01	0.99 ± 0.01	0.79 ± 0.01
RF	0.73 ± 0.01	0.75 ± 0.041	0.90 ± 0.01	0.72 ± 0.01
XGBoost	0.73 ± 0.01	0.77 ± 0.01	0.82 ± 0.02	0.79 ± 0.01
SVM	0.71 ± 0.01	0.81 ± 0.01	0.71 ± 0.02	0.75 ± 0.01
NN	0.65 ± 0.02	0.72 ± 0.01	0.74 ± 0.02	0.73 ± 0.02
LightGBM	0.71 ± 0.01	0.79 ± 0.01	0.76 ± 0.02	0.77 ± 0.01
Catboost	0.72 ± 0.02	0.79 ± 0.01	0.77 ± 0.02	0.78 ± 0.02

### 3.5 Precision–recall curve analysis for prediction of weight loss/improvement

**Figure 4:**
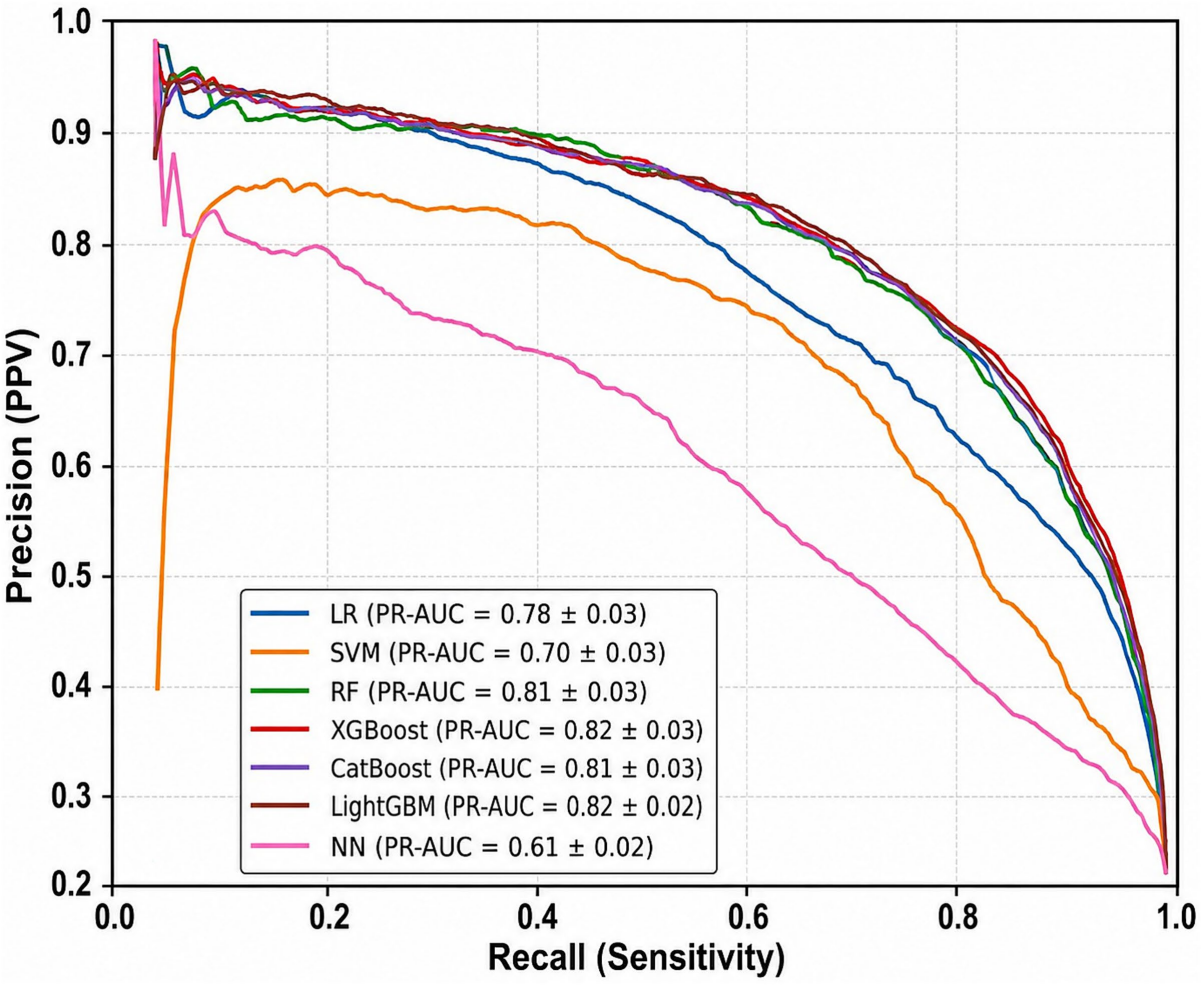
10-fold cross-validated precision–recall curves for prediction of weight loss/improvement following GLP-1 RA Therapy.Precision–recall (PR) curves showing the performance of machine learning models for predicting clinically meaningful weight improvement following GLP-1 RA therapy. Curves are based on 10-fold cross-validation. The x-axis represents recall (sensitivity), and the y-axis represents precision (positive predictive value). PR-AUC values are reported as mean ± standard deviation. Ensemble models demonstrated the highest precision–recall performance, indicating improved ability to identify true responders while minimizing false-positive predictions. These findings support the potential utility of machine learning models for patient stratification and prediction of treatment response in obesity management.

The precision–recall (PR) curve analysis demonstrated consistent differences in predictive performance across machine learning models for identifying individuals who achieved clinically meaningful weight improvement following GLP-1 RA therapy. Overall, tree-based ensemble models exhibited the strongest performance with XGBoost and LightGBM achieved the highest PR-AUC values (0.82 ± 0.03 and 0.82 ± 0.02, respectively). In contrast, the NN model exhibited the lowest predictive performance (PR-AUC: 0.61 ± 0.02), indicating reduced reliability in distinguishing responders from non-responders (Figure 4).

### 3.6 Precision–recall curve analysis for glycemic control prediction

**Figure 5:**
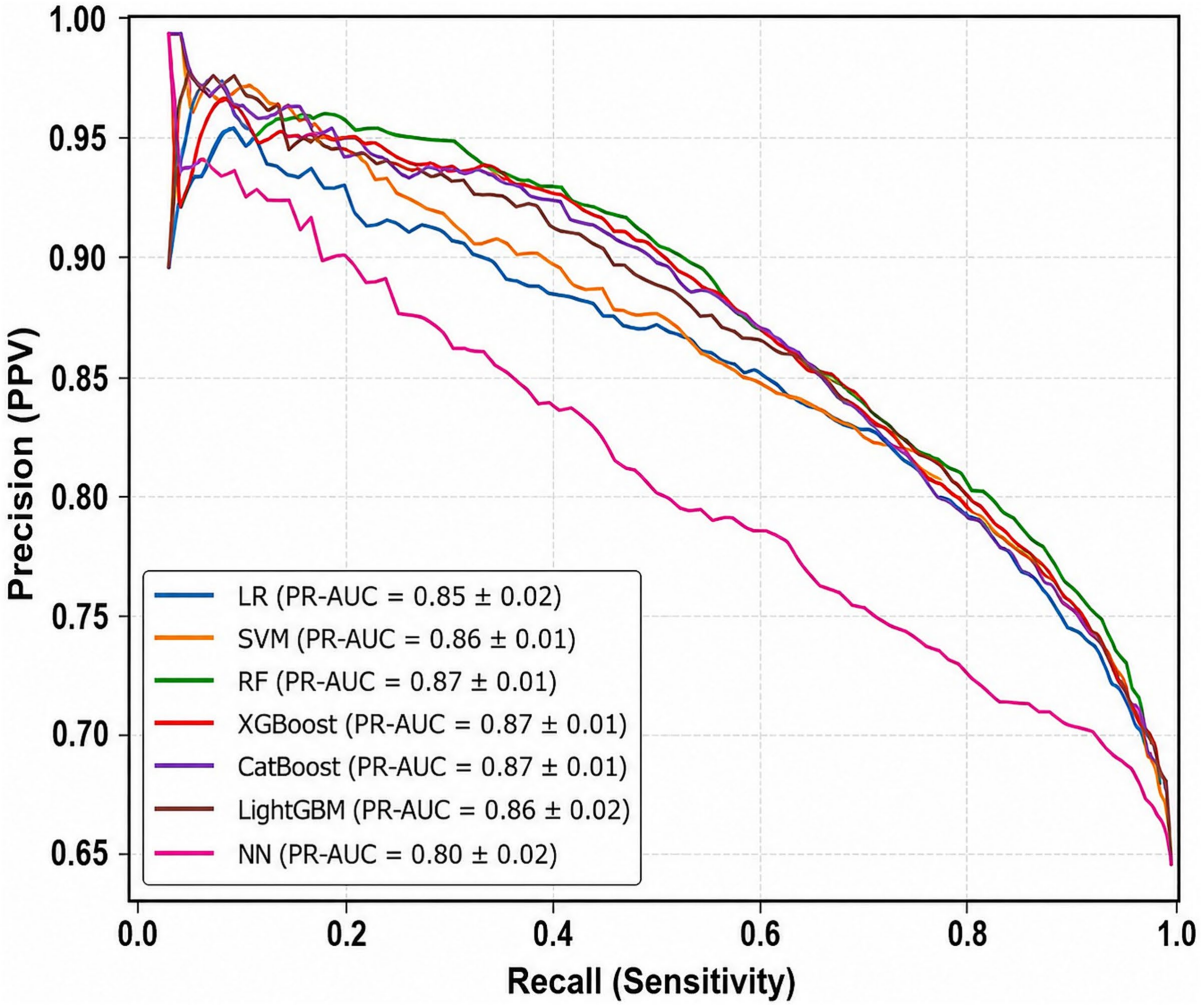
10-fold cross-validated precision–recall curves for prediction of glycemic control following GLP-1 RA therapy.Precision–recall (PR) curves showing the performance of machine learning models for predicting optimal glycemic control following GLP-1 RA therapy. Curves are based on 10-fold cross-validation. The x-axis represents recall (sensitivity), and the y-axis represents precision (positive predictive value). PR-AUC values are reported as mean ± standard deviation. Tree-based ensemble models maintained high precision across a broad range of recall values, indicating stable performance in identifying likely responders while reducing false-positive classifications. These findings suggest that machine learning approaches may assist in identifying patients most likely to benefit from GLP-1 RA therapy.

The precision–recall (PR) curve analysis demonstrated strong and relatively consistent predictive performance across machine learning models for identifying individuals achieving optimal glycemic control following GLP-1 RA therapy. Tree-based ensemble models, including RF, XGBoost, and CatBoost, achieved the highest performance (PR-AUC ≈ 0.87 ± 0.01), followed by LightGBM (PR-AUC: 0.86 ± 0.02). These models maintained high precision across a wide range of recall values, indicating stable performance in identifying responders while minimizing false-positive predictions (Figure 5).

### 3.7 Explainable machine learning identification of predictors of weight improvement following GLP-1 RA therapy

**Figure 6:**
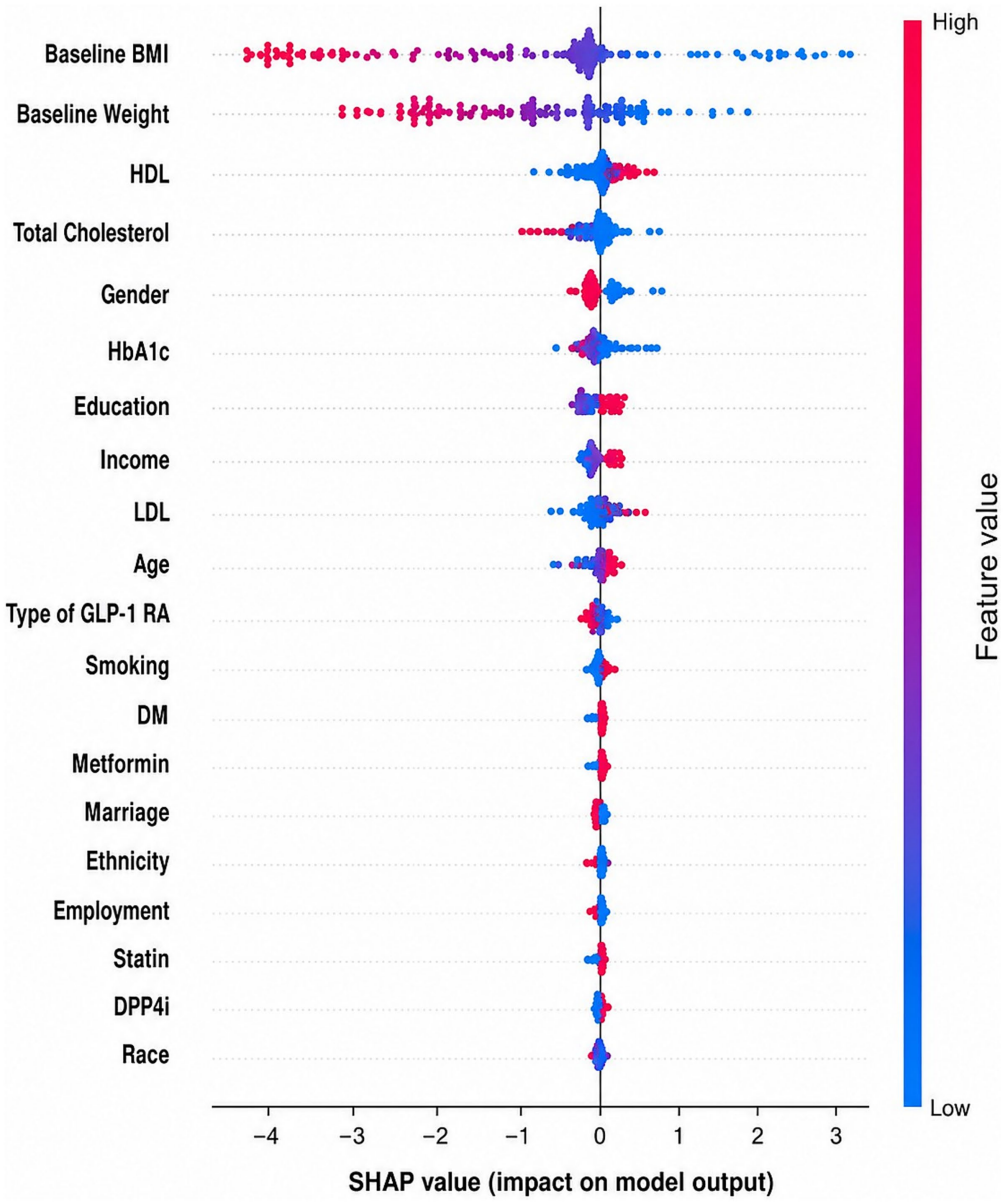
SHAP summary plot of predictors of weight loss/improvement following GLP-1 RA Therapy.SHAP summary plot showing the contribution of each feature to the prediction of weight improvement following GLP-1 RA therapy. Each point represents an individual observation. The x-axis indicates SHAP values, where positive values correspond to a higher likelihood of weight improvement and negative values indicate a lower likelihood. Features are ordered by mean absolute SHAP importance. Color represents feature values (red = higher values; blue = lower values). Baseline BMI and body weight emerged as the most influential predictors, suggesting that baseline anthropometric characteristics play a major role in determining treatment response. Higher HDL cholesterol levels were also associated with improved outcomes, potentially reflecting better underlying metabolic health. Importantly, SHAP values represent predictive importance within the model and should not be interpreted as evidence of causal relationships.

SHAP analysis identified baseline anthropometric measures as the dominant predictors of weight improvement following GLP-1 RA therapy. In particular, baseline BMI and baseline weight showed the largest magnitude of contribution, with lower values consistently associated with a higher likelihood of achieving weight improvement. Among metabolic indicators, HDL cholesterol and total cholesterol demonstrated moderate contributions, while HbA1c showed a relatively smaller but still noticeable effect. Higher HDL levels were generally associated with improved outcomes. In contrast, several categorical variables, including race, ethnicity, employment status, marital status,—showed minimal impact (Figure 6).

### 3.8 Explainable machine learning identification of predictors of glycemic control following GLP-1 RA therapy

**Figure 7:**
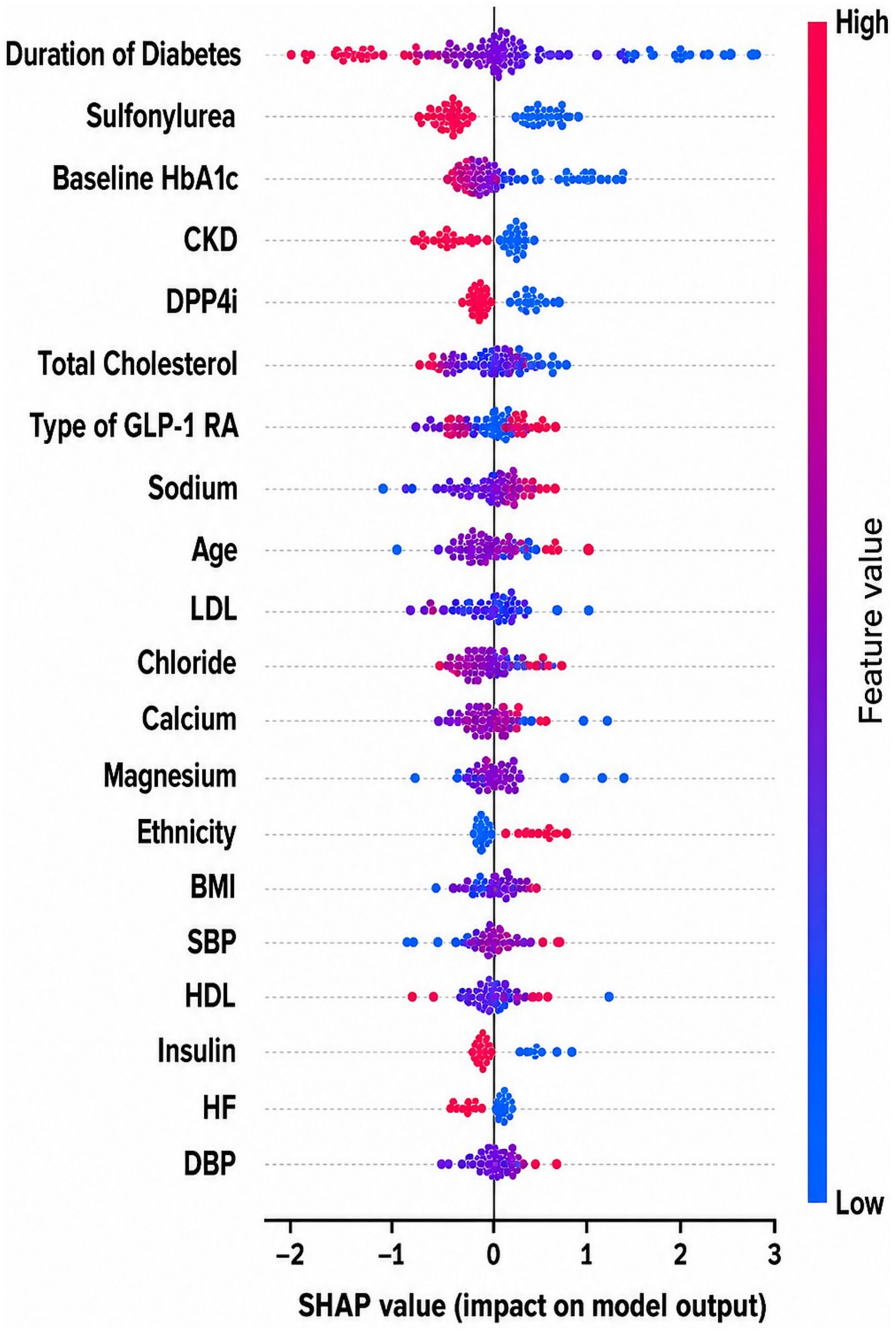
Explainable machine learning identification of predictors of glycemic control following GLP-1 RA Therapy.SHAP summary plot showing feature contributions to the prediction of glycemic control following GLP-1 RA therapy. Each point represents an individual observation. The x-axis indicates SHAP values, where positive values correspond to a higher likelihood of glycemic control and negative values indicate a lower likelihood. Features are ordered by mean absolute SHAP importance. Color represents feature magnitude (red = higher values; blue = lower values). Duration of diabetes, baseline HbA1c, sulfonylurea use, and insulin use were among the most influential predictors, indicating that disease severity and treatment complexity are strongly associated with glycemic response. These findings may help identify patient characteristics associated with treatment success; however, SHAP values represent predictive associations rather than causal effects.

SHAP analysis identified duration of diabetes as the most influential predictor of glycemic control, followed by sulfonylurea use and baseline HbA1c. Longer diabetes duration and higher baseline HbA1c were strongly associated with a lower likelihood of achieving glycemic control, whereas shorter duration and lower baseline HbA1c were associated with improved outcomes, reflecting the impact of underlying disease severity on treatment response. Treatment-related factors such as sulfonylurea and insulin use was associated with reduced likelihood of glycemic control, consistent with more advanced or treatment-resistant disease. The type of GLP-1 RA exhibited modest influence, with small variability across agents but no dominant effect (Figure 7).

### 3.9 Sensitivity analysis

We conducted a sensitivity analysis using a clinically meaningful weight-loss outcome defined as ≥5% reduction from baseline body weight as an outcome variable. Predictive performance was attenuated compared with the primary BMI-based outcome (BMI < 30 kg/m^2^ at follow-up). However, tree-based ensemble models continued to demonstrate acceptable and relatively robust performance. RF and XGBoost models achieved the highest AUC score (0.72 ± 0.01) (Supplementary Table S3).

As an additional sensitivity analysis, baseline BMI was excluded from the predictor set while retaining the original BMI-based outcome definition to evaluate whether model performance was influenced by the mathematical relationship between baseline BMI and the primary outcome definition. The findings showed that, although predictive performance declined compared with the primary analysis, several machine learning models maintained acceptable performance (Supplementary Table S4). The RF and XGBoost models achieved the highest AUC (0.90 ± 0.01). These findings suggest that model performance was not solely driven by mathematical coupling between baseline BMI and follow-up BMI but also reflected clinically meaningful predictors of treatment response.

For glycemic control prediction, baseline HbA1c was excluded from the predictor set to evaluate its influence on model performance. Overall, predictive performance showed a slight decline across all machine learning models compared with the primary analysis, confirming the importance of baseline HbA1c as a predictor of glycemic control. Nevertheless, several models maintained acceptable predictive performance. The XGBoost model achieved the highest overall performance (AUC: 0.79 ± 0.01), followed by the RF model (0.78 ± 0.011) (Supplementary Table S5). These findings suggest that although baseline HbA1c important feature of glycemic control, other factors continue to provide substantial predictive information regarding treatment response following GLP-1 RA therapy (Supplementary Table S5).

In addition to discrimination metrics, we further evaluated model calibration performance using the Brier score, which measures the accuracy of probabilistic predictions. The ML models demonstrated an average Brier score of 0.08 for the weight loss outcome prediction and 0.18 for the glycemic control prediction model, indicating acceptable overall probabilistic prediction accuracy, with stronger calibration performance observed for the weight loss outcome models.

## 4 Discussion

In this study, we evaluated multiple supervised machine learning algorithms to predict weight loss outcomes and glycemic control following GLP-1 RA therapy using real-world data. Overall, tree-based ensemble models consistently demonstrated superior predictive performance compared with traditional and non-ensemble approaches. For weight loss prediction, ensemble models—including RF, XGBoost, LightGBM, and CatBoost—achieved the highest discrimination and classification performance. In particular, RF and XGBoost demonstrated the strongest overall performance, with AUC values of approximately 0.94 and high accuracy (0.89–0.90).

For glycemic control prediction, overall model performance was more modest compared to weight loss prediction, reflecting the greater complexity and heterogeneity of glycemic outcomes. Nevertheless, RF and XGBoost again demonstrated relatively strong and balanced performance across evaluation metrics. Both models achieved similar accuracy (~0.73), while RF showed higher sensitivity and XGBoost demonstrated a more balanced trade-off between precision and recall.

Tree-based ensemble models are well known for their ability to model complex nonlinear relationships, automatically capture higher-order interactions, and handle heterogeneous data types without extensive feature engineering (Hu and Li, 2022). Previous studies have similarly reported improved predictive performance of boosting algorithms in clinical prediction tasks. For example, a study developing a machine learning model to predict weight loss success in a behavioral intervention trial demonstrated that RF-based models improved predictive accuracy and generalizability compared with traditional clinical decision rules (Shahabi et al., 2024). Similarly, other investigations have highlighted the versatility of tree-based methods in healthcare research, including their application in risk prediction, causal inference, propensity score estimation, and missing data imputation. These capabilities make them particularly well suited for analyzing high-dimensional real-world datasets, where complex relationships among predictors are common (Hu and Li, 2022; Stahl, 2024). In the context of glycemic control prediction, our findings are also aligned with prior studies demonstrating strong performance of tree-based ensemble models in predicting blood glucose levels and glycemic outcomes in patients with diabetes (Fu et al., 2023; Ali et al., 2024). These models have shown robustness across different datasets and clinical settings, including real-time glucose prediction and long-term glycemic control estimation (Cai et al., 2025; Van Doorn et al., 2021).

However, not all studies have found ensemble methods to be superior. For instance, some studies in bariatric surgery populations reported that SVM achieved the highest predictive performance, suggesting that model performance may depend on the underlying data structure, feature selection, and patient population characteristics (Casas Domínguez et al., 2025). Differences in predictor variables—such as inclusion of biochemical markers, behavioral factors, or disease-specific variables—as well as differences in cohort composition and outcome definitions may explain these inconsistencies across studies (Nadal et al., 2024; Glasbrenner et al., 2024; Biehl et al., 2023).

In addition to predictive performance, this study provides clinically meaningful insights through explainable machine learning using SHAP analysis, highlighting key patient characteristics features associated with both weight and glycemic outcomes following GLP-1 RA therapy. For weight loss outcome, baseline anthropometric measures—particularly BMI and body weight—emerged as the most important features, with lower baseline values consistently associated with a higher reduction in weight. These findings suggest that individuals with lower baseline adiposity may have a more favorable metabolic profile, including reduced insulin resistance and inflammation, thereby enhancing responsiveness to GLP-1 RA therapy (Burhans et al., 2019). These findings are partially consistent with prior literature but also highlight important differences. For example, a meta-analysis of randomized controlled trials reported that greater weight loss benefits were observed among individuals with higher baseline BMI and weight values (Wong et al., 2025). Similarly, some real-world studies have shown that baseline BMI is not always independently associated with weight loss response (Squire et al., 2025). This discrepancy likely reflects differences in obesity subtypes, interventions, and population characteristics that might influence treatment success (Vozza et al., 2025; Nathan et al., 2016). Additionally, HDL cholesterol demonstrated moderate importance, with higher levels associated with improved weight outcomes. This likely reflects underlying metabolic health, as higher HDL is associated with improved insulin sensitivity, lower systemic inflammation, and more efficient lipid metabolism, all of which facilitate weight reduction (Stadler and Marsche, 2020).

For glycemic control, SHAP analysis identified that longer duration of diabetes appeared to be associated with poor glycemic control. This finding is consistent with prior studies demonstrating that glycemic control worsens with increasing disease duration due to progressive beta-cell dysfunction and worsening insulin resistance (Verma et al., 2006). In addition, longer disease duration is often associated with increased comorbidity burden and treatment complexity, further limiting the ability to achieve optimal glycemic outcomes (Kassie et al., 2025). Treatment-related factors, including sulfonylurea and insulin use, were associated with reduced likelihood of glycemic control. These therapies are often markers of more advanced or treatment-resistant disease, primarily address insulin deficiency rather than underlying insulin resistance, and their long-term effectiveness may be limited by progressive beta-cell decline (Insulin, 2023).

Nonetheless, the SHAP analyses performed in this study identify the relative predictive contribution of features within the machine learning models and should not be interpreted as evidence of causal relationships. Rather, SHAP values reflect associative predictive importance based on the patterns learned from the training data. Consequently, predictors identified as highly influential may represent markers of disease severity, treatment complexity, or correlated clinical characteristics rather than direct mechanistic determinants of treatment response. Despite this limitation, the explainable machine learning framework provides clinically relevant insights into patient-level factors associated with glycemic and weight outcomes following GLP-1 RA therapy. These predictive models may help support patient risk stratification and individualized treatment planning by identifying individuals who are more or less likely to achieve favorable treatment responses. In addition, explainable ML approaches may assist clinicians in recognizing clinically important characteristics associated with treatment response and may help guide earlier monitoring or therapeutic optimization strategies. However, these applications remain exploratory and require prospective external validation before implementation in routine clinical decision-making workflows.

### 4.1 Strengths and limitations

This study has several important strengths. First, it used data from the AoU Research Program, a large and diverse national dataset with substantial representation of populations historically underrepresented in biomedical research. Second, the study evaluated multiple machine learning algorithms using a consistent analytic framework, allowing robust comparison of model performance across traditional and ensemble approaches. Finally, the application of SHAP improved model interpretability by identifying predictors of treatment response, thereby enhancing the translational value of the machine learning findings.

This study also has limitations. First, the study measured follow-up outcomes within a relatively short follow-up period, which may not fully capture longer-term glycemic and weight trajectories after GLP-1 RA initiation. Second, treatment adherence, dose escalation, medication persistence, lifestyle interventions, and provider-level factors were not fully captured, although these factors likely influence both glycemic and weight outcomes. Third, HbA1c levels may vary across racial and ethnic populations independent of mean glucose levels, which may influence interpretation of glycemic control outcomes. Although the models demonstrated strong internal performance, the current findings should be interpreted cautiously because external validation was not performed. The predictive models were developed and internally validated within the All of Us Research Program dataset and therefore require validation in independent healthcare systems and clinical populations before implementation in routine clinical practice. In addition, while SHAP analysis improved explainability of the machine learning models, identified predictors should not be interpreted as causal determinants of treatment response.

### 4.2 Future directions

Future research should externally validate these models using independent clinical and population-based datasets to assess their transportability, generalizability, and real-world applicability across diverse healthcare settings. Following external validation, future studies should also explore the development of a clinical decision-support tool and evaluate its feasibility, usability, and implementation in clinical practice. Longer follow-up periods are also needed to evaluate sustained glycemic control, long-term weight change, treatment discontinuation, and adverse events after GLP-1 RA initiation. Incorporating additional longitudinal and time-updated features, such as medication adherence, dose changes, treatment switching, and laboratory trends, may further improve predictive performance and better reflect the dynamic nature of treatment response.

## 5 Conclusion

In conclusion, machine learning models demonstrated meaningful potential for predicting glycemic control and weight improvement following GLP-1 RA therapy using real-world data from a large and diverse national cohort. Tree-based ensemble methods consistently showed acceptable predictive performance, suggesting that these methods are better able to capture the complex and nonlinear relationships underlying treatment response. Explainable machine learning further showed that anthropometric measures were central determinants of weight improvement whereas duration of diabetes, baseline HbA1c, and treatment-related factors were key drivers of glycemic control.

## Data Availability

The original contributions presented in the study are included in the article/Supplementary material, further inquiries can be directed to the corresponding author/s.

## Glossary

AoU: All of Us Research Program.
AUC: Area Under the Curve.
BMI: Body Mass Index.
CKD: Chronic Kidney Disease.
DBP: Diastolic Blood Pressure.
DPP-4i: Dipeptidyl Peptidase-4 Inhibitor.
EHR: Electronic Health Record.
F1-score: F1 Score.
GLP-1: RA Glucagon-Like Peptide-1 Receptor Agonist.
HbA1c: Hemoglobin A1c.
HDL: High-Density Lipoprotein.
HF: Heart Failure.
LDL: Low-Density Lipoprotein.
LightGBM: Light Gradient Boosting Machine.
LR: Logistic Regression.
ML: Machine Learning.
NN: Neural Network.
PR-AUC: Area Under the Precision–Recall Curve.
RF: Random Forest.
ROC: Receiver Operating Characteristic.
SBP: Systolic Blood Pressure.
SHAP: SHapley Additive exPlanations.
SGLT2i: Sodium-Glucose Cotransporter-2 Inhibitor.
SVM: Support Vector Machine.
T2DM: Type 2 Diabetes Mellitus.

## References

[ref1] Abualigah L. Alomari S. A. Almomani M. H. Zitar R. A. Saleem K. Migdady H. . (2025). Artificial intelligence-driven translational medicine: a machine learning framework for predicting disease outcomes and optimizing patient-centric care. J. Transl. Med. 23:302. doi: 10.1186/s12967-025-06308-6, 40065389 PMC11892274

[ref2] Ali H. Niazi I. K. White D. Akhter M. N. Madanian S. (2024). Comparison of machine learning models for predicting interstitial glucose using smart watch and food log. Electronics 13:3192. doi: 10.3390/electronics13163192

[ref3] Alowais S. A. Alghamdi S. S. Alsuhebany N. Alqahtani T. Alshaya A. I. Almohareb S. N. . (2023). Revolutionizing healthcare: the role of artificial intelligence in clinical practice. BMC Med. Educ. 23:689. doi: 10.1186/s12909-023-04698-z, 37740191 PMC10517477

[ref4] Amer Diabet Assoc Professional Practice Comm NA Elsayed N. A. Mccoy R. G. Aleppo G. Balapattabi K. Beverly E. A. . (2025). 6. Glycemic goals and hypoglycemia: standards of care in diabetes-2025. Diabetes Care 48, S128–S145. doi: 10.2337/dc25-S006, 39651981 PMC11635034

[ref5] AoURP I. (2019). The “all of us” research program. N. Engl. J. Med. 381, 668–676. doi: 10.1056/NEJMsr1809937, 31412182 PMC8291101

[ref6] Azur M. J. Stuart E. A. Frangakis C. Leaf P. J. (2011). Multiple imputation by chained equations: what is it and how does it work? Int. J. Methods Psychiatr. Res. 20, 40–49. doi: 10.1002/mpr.329, 21499542 PMC3074241

[ref7] Biehl A. Venäläinen M. S. Suojanen L. U. Kupila S. Ahola A. J. Pietiläinen K. H. . (2023). Development and validation of a weight-loss predictor to assist weight loss management. Sci. Rep. 13:20661. doi: 10.1038/s41598-023-47930-y, 38001145 PMC10673897

[ref8] Burhans M. S. Hagman D. K. Kuzma J. N. Schmidt K. A. Kratz M. (2019). Contribution of adipose tissue inflammation to the development of type 2 diabetes mellitus. Compr. Physiol. 9, 1–58. doi: 10.1002/j.2040-4603.2019.tb00055.x PMC6557583 30549014

[ref9] Cai S. Hu Y. Hong Y. Qian L. Lin S. Lin X. . (2025). Development and validation of a machine learning model for real-time blood glucose prediction for ICU patients. BMC Med. Inform. Decis. Mak. 26:14. doi: 10.1186/s12911-025-03309-9, 41366380 PMC12801964

[ref10] Casas Domínguez M. Herrena Montano I. López Gómez J. J. Ramos Bachiller B. de Luis Román D. A. IdlT D. (2025). Predicting weight loss success after gastric sleeve surgery: a machine learning-based approach. Nutrients 17, 1–13. doi: 10.3390/nu17081391, 40284254 PMC12030315

[ref11] Connery H. S. McHugh R. K. Reilly M. Shin S. Greenfield S. F. (2020). Substance use disorders in global mental health delivery: epidemiology, treatment gap, and implementation of evidence-based treatments. Harv. Rev. Psychiatry 28, 316–327. doi: 10.1097/HRP.0000000000000271, 32925514 PMC8324330

[ref12] Dawed A. Y. Mari A. Brown A. McDonald T. J. Li L. Wang S. . (2023). Pharmacogenomics of GLP-1 receptor agonists: a genome-wide analysis of observational data and large randomised controlled trials. Lancet Diabetes Endocrinol. 11, 33–41. doi: 10.1016/S2213-8587(22)00340-0, 36528349

[ref13] Franch-Nadal J. Granado-Casas M. Mata-Cases M. Ortega E. Vlacho B. Mauricio D. (2022). Determinants of response to the glucagon-like peptide-1 receptor agonists in a type 2 diabetes population in the real-world. Prim. Care Diabetes 16, 810–817. doi: 10.1016/j.pcd.2022.10.005, 36336605

[ref14] Fu X. Wang Y. Cates R. S. Li N. Liu J. Ke D. . (2023). Implementation of five machine learning methods to predict the 52-week blood glucose level in patients with type 2 diabetes. Front. Endocrinol. 13:1061507. doi: 10.3389/fendo.2022.1061507, 36743935 PMC9895792

[ref15] Glasbrenner C. Höchsmann C. Pieper C. F. Wasserfurth P. Dorling J. L. Martin C. K. . (2024). Prediction of individual weight loss using supervised learning: findings from the CALERIETM 2 study. Am. J. Clin. Nutr. 120, 1233–1244. doi: 10.1016/j.ajcnut.2024.09.003, 39270937 PMC11600119

[ref16] Hu L. Li L. (2022). Using tree-based machine learning for health studies: literature review and case series. Int. J. Environ. Res. Public Health 19:16080. doi: 10.3390/ijerph192316080, 36498153 PMC9736500

[ref17] Insulin F. A. (2023). “Resistance StatPearls. Treasure Island FL ineligible companies. Disclosure: Luis Acevedo declares no relevant financial relationships with ineligible companies,” In: Freeman AM, Acevedo LA, Pennings N. Eds., and Insulin Resistance. StatPearls [Internet]. Treasure Island, FL: StatPearls Publishing LLC. Available online at: https://www.ncbi.nlm.nih.gov/books/NBK507839/.

[ref18] The All of Us Research Program Genomics Investigators (2024). Genomic data in the All of Us Research Program. Nature. 627, 340–348. doi: 10.1038/s41586-023-06957-x 38374255 PMC10937371

[ref19] Kassie M. Z. Alemu C. Wudu H. Marine B. T. Gebeyehu A. A. (2025). Time to achieve optimal glycemic control and its determinants among diabetes mellitus patients receiving treatment: a retrospective study. Sci. Rep. 15:20031. doi: 10.1038/s41598-025-96097-1, 40481244 PMC12144107

[ref20] Khalifa M. Albadawy M. (2024). Artificial intelligence for clinical prediction: exploring key domains and essential functions. Computer Methods Programs Biomedicine Update. 5:100148. doi: 10.1016/j.cmpbup.2024.100148

[ref21] Lundberg S. M. Lee S.-I. (2017). A unified approach to interpreting model predictions. In: Guyon I, von Luxburg U, Bengio S, Wallach H, Fergus R, Vishwanathan S, and Garnett R, editors. Advances in Neural Information Processing Systems 30 (NeurIPS 2017). Red Hook, NY: Curran Associates, Inc. 4765–4774.

[ref22] Marees A. T. De Kluiver H. Stringer S. Vorspan F. Curis E. Marie-Claire C. . (2018). A tutorial on conducting genome-wide association studies: quality control and statistical analysis. Int. J. Methods Psychiatr. Res. 27:e1608. doi: 10.1002/mpr.1608, 29484742 PMC6001694

[ref23] Nadal E. Benito E. Ródenas-Navarro A. M. Palanca A. Martinez-Hervas S. Civera M. . (2024). Machine learning model in obesity to predict weight loss one year after bariatric surgery: a pilot study. Biomedicine 12:1175. doi: 10.3390/biomedicines12061175, 38927382 PMC11200726

[ref24] Nathan B. M. Rudser K. D. Abuzzahab M. J. Fox C. K. Coombes B. J. Bomberg E. M. . (2016). Predictors of weight-loss response with glucagon-like peptide-1 receptor agonist treatment among adolescents with severe obesity. Clinical obesity. 6, 73–78. doi: 10.1111/cob.12128, 26683756 PMC4721217

[ref25] Norbury A. Seymour B. (2018). Response heterogeneity: challenges for personalised medicine and big data approaches in psychiatry and chronic pain. F1000Res. 7:55. doi: 10.12688/f1000research.13723.2, 29527298 PMC5820606

[ref26] Rainio O. Teuho J. Klén R. (2024). Evaluation metrics and statistical tests for machine learning. Sci. Rep. 14:6086. doi: 10.1038/s41598-024-56706-x, 38480847 PMC10937649

[ref27] Rajula H. S. R. Verlato G. Manchia M. Antonucci N. Fanos V. (2020). Comparison of conventional statistical methods with machine learning in medicine: diagnosis, drug development, and treatment. Medicina 56:455. doi: 10.3390/medicina56090455, 32911665 PMC7560135

[ref28] Ramirez A. H. Sulieman L. Schlueter D. J. Halvorson A. Qian J. Ratsimbazafy F. . (2022). The All of Us Research Program: data quality, utility, and diversity. Patterns. 3:100570. doi: 10.1016/j.patter.2022.100570, 36033590 PMC9403360

[ref29] Rao P. P. (2026). Revolutionizing obesity treatment: the emerging role of GLP-1 receptor agonists and next-generation pharmacotherapies. Obesity Medicine. 60:100686. doi: 10.1016/j.obmed.2026.100686, 38826717

[ref30] Shahabi F. Battalio S. L. Pfammatter A. F. Hedeker D. Spring B. Alshurafa N. (2024). A machine-learned model for predicting weight loss success using weight change features early in treatment. NPJ digital medicine. 7:344. doi: 10.1038/s41746-024-01299-y, 39613928 PMC11607303

[ref31] Squire P. Naude J. Zentner A. Bittman J. Khan N. (2025). Factors associated with weight loss response to GLP-1 analogues for obesity treatment: a retrospective cohort analysis. BMJ Open 15:e089477. doi: 10.1136/bmjopen-2024-089477, 39819958 PMC11751938

[ref32] Stadler J. T. Marsche G. (2020). Obesity-related changes in high-density lipoprotein metabolism and function. Int. J. Mol. Sci. 21:8985. doi: 10.3390/ijms21238985, 33256096 PMC7731239

[ref33] Stahl D. (2024). New horizons in prediction modelling using machine learning in older people’s healthcare research. Age Ageing 53:afae201. doi: 10.1093/ageing/afae201, 39311424 PMC11417961

[ref34] Swift D. L. Johannsen N. M. Lavie C. J. Earnest C. P. Blair S. N. Church T. S. (2016). Effects of clinically significant weight loss with exercise training on insulin resistance and cardiometabolic adaptations. Obesity 24, 812–819. doi: 10.1002/oby.21404, 26935138 PMC4814330

[ref35] Thomas D. M. Ivanescu A. E. Martin C. K. Heymsfield S. B. Marshall K. Bodrato V. E. . (2015). Predicting successful long-term weight loss from short-term weight-loss outcomes: new insights from a dynamic energy balance model (the POUNDS lost study). Am. J. Clin. Nutr. 101, 449–454. doi: 10.3945/ajcn.114.091520, 25733628 PMC4340057

[ref36] Van Doorn W. P. Foreman Y. D. Schaper N. C. Savelberg H. H. Koster A. van der Kallen C. J. . (2021). Machine learning-based glucose prediction with use of continuous glucose and physical activity monitoring data: the Maastricht study. PLoS One 16:e0253125. doi: 10.1371/journal.pone.0253125, 34166426 PMC8224858

[ref37] Verma M. Paneri S. Badi P. Raman P. (2006). Effect of increasing duration of diabetes mellitus type 2 on glycated hemoglobin and insulin sensitivity. Indian J. Clin. Biochem. 21, 142–146. doi: 10.1007/BF02913083, 23105586 PMC3453763

[ref38] Vozza A. Triggiani D. Fanelli M. Lisco G. Coletto D. Custodero C. . (2025). Predictive factors of body weight loss in patients with type 2 diabetes treated with GLP-1 receptor agonists: a 52-week prospective real-life study. Front. Endocrinol. 16:1674308. doi: 10.3389/fendo.2025.1674308, 41079186 PMC12507565

[ref39] Wong H. J. Sim B. Teo Y. H. Teo Y. N. Chan M. Y. Yeo L. L. . (2025). Efficacy of GLP-1 receptor agonists on weight loss, BMI, and waist circumference for patients with obesity or overweight: a systematic review, meta-analysis, and meta-regression of 47 randomized controlled trials. Diabetes Care 48, 292–300. doi: 10.2337/dc24-1678 39841962

[ref40] Zheng Z. Zong Y. Ma Y. Tian Y. Pang Y. Zhang C. . (2024). Glucagon-like peptide-1 receptor: mechanisms and advances in therapy. Signal Transduct. Target. Ther. 9:234. doi: 10.1038/s41392-024-01931-z, 39289339 PMC11408715

