# Supplementary material for: Machine learning algorithms for predicting glycemic control and weight loss outcomes in GLP-1 receptor agonist users
Source: Front Artif Intell. 2026 Jul 15;9:1861563. doi: 10.3389/frai.2026.1861563 (PMC13416677; doi:10.3389/frai.2026.1861563)
Supplement: Supplementary file 1 [file Data_Sheet_1.pdf]

Supplementary File Tables

Table S1: Baseline Characteristics of Participants in the Weight Loss Cohort

Table S2: Baseline Characteristics of Participants in the Glycemic Control Cohort

Table S3: Sensitivity Analysis Using ≥5% Weight Loss Threshold

Table S4: Sensitivity Analysis After Excluding Baseline BMI from Weight Prediction Models

Table S5: Sensitivity Analysis After Excluding Baseline HbA1c from Glycemic Control Prediction Models

Table S1: Baseline Characteristics of Participants in the Weight Loss Cohort

**Variable**	**Category**	**Not Improved (n=9,004)**	**Improved (n=2,416)**	**Total (n=11,420)**
**Gender**	Female	6023 (52.8%)	1422 (12.5%)	7445 (65.2%)
	Male	2847 (24.9%)	956 (8.4%)	3803 (33.3%)
	Others	134 (1.2%)	38 (0.3%)	172 (1.5%)
**Race**	White	4699 (41.1%)	1167 (10.2%)	5866 (51.4%)
	Black	2000 (17.5%)	438 (3.8%)	2438 (21.3%)
	Asian	120 (1.1%)	98 (0.9%)	218 (1.9%)
	Others	2185 (19.1%)	713 (6.2%)	2898 (25.4%)
**Ethnicity**	Non-Hispanic	7213 (63.2%)	1786 (15.6%)	8999 (78.8%)
	Hispanic	1541 (13.5%)	552 (4.8%)	2093 (18.3%)
	Other	250 (2.2%)	78 (0.7%)	328 (2.9%)
**GLP-1 RA Type**	Semaglutide	3595 (31.5%)	924 (8.1%)	4519 (39.6%)
	Dulaglutide	2362 (20.7%)	750 (6.6%)	3112 (27.3%)
	Exenatide	2371 (20.8%)	578 (5.1%)	2949 (25.8%)
	Liraglutide	603 (5.3%)	147 (1.3%)	750 (6.6%)

	Other	73 (0.6%)	17 (0.1%)	90 (0.8%)
**Education**	No high school	3445 (30.2%)	847 (7.4%)	4292 (37.6%)
	High school	2075 (18.2%)	491 (4.3%)	2566 (22.5%)
	College graduate	3484 (30.5%)	1078 (9.4%)	4562 (39.9%)
**Insurance**	Insured	8611 (75.4%)	2306 (20.2%)	10917 (95.6%)
**Employment**	Employed	3549 (31.1%)	812 (7.1%)	4361 (38.2%)
**Income**	<25k	2295 (20.1%)	525 (4.6%)	2820 (24.7%)
	25k–50k	1603 (14.0%)	352 (3.1%)	1955 (17.1%)
	50k–100k	2015 (17.6%)	459 (4.0%)	2474 (21.7%)
	100k–200k	1251 (11.0%)	404 (3.5%)	1655 (14.5%)
	>200k	1840 (16.1%)	676 (5.9%)	2516 (22.0%)
**Marital Status**	Married	3914 (34.3%)	1152 (10.1%)	5066 (44.4%)
**Medications**	Sulfonylurea	2808 (24.6%)	931 (8.2%)	3739 (32.7%)
	Insulin	6144 (53.8%)	1708 (15.0%)	7852 (68.8%)
	SGLT2i	2759 (24.2%)	889 (7.8%)	3648 (31.9%)
	Metformin	6215 (54.4%)	1739 (15.2%)	7954 (69.6%)
	DPP4i	3935 (34.5%)	1307 (11.4%)	5242 (45.9%)
	Statin	6245 (54.7%)	1873 (16.4%)	8118 (71.1%)
**Lifestyle**	Alcohol use	7861 (68.8%)	2071 (18.1%)	9932 (87.0%)
	Smoking	3671 (32.1%)	990 (8.7%)	4661 (40.8%)
**Comorbidities**	Diabetes	6610 (57.9%)	1912 (16.7%)	8522 (74.6%)
	CKD	6610 (57.9%)	1912 (16.7%)	8522 (74.6%)
	Heart failure	1782 (15.6%)	432 (3.8%)	2214 (19.4%)

**Continuous Variables (Mean ± SD)**	Age (years)	54.07 ± 12.46	58.96 ± 12.06	55.11 ± 12.53
	HbA1c (%)	7.15 ± 1.79	7.40 ± 1.59	7.20 ± 1.75
	HDL	46.31 ± 11.51	48.71 ± 12.98	46.82 ± 11.88
	LDL	96.55 ± 28.44	94.85 ± 29.16	96.19 ± 28.60
	Total cholesterol	175.15 ± 33.56	175.88 ± 35.36	175.30 ± 33.95
	Baseline weight (kg)	112.79 ± 26.77	81.70 ± 14.60	106.21 ± 27.77
	Baseline BMI (kg/m²)	39.41 ± 7.32	29.95 ± 4.67	37.41 ± 7.86

Table S1 shows how participants were classified according to whether they achieved the primary weight outcome, defined as a follow-up BMI <30 kg/m² after GLP-1 receptor agonist initiation. Categorical variables are presented as frequencies and percentages, and continuous variables are presented as mean ± standard deviation (SD). Percentages are calculated based on the total study population (N = 11,420).

Table S2: Baseline Characteristics of Participants in the Glycemic Control Cohort

Variable	Category	Uncontrolled (n=1426)	Controlled (n=2,549)	Total (N=3975)
**Gender**	Female	655 (16.5%)	1109 (27.9%)	1764 (44.4%)
	Male	753 (18.9%)	1409 (35.4%)	2162 (54.4%)
	Other	18 (0.5%)	31 (0.8%)	49 (1.2%)
**Race**	White	761 (19.2%)	1420 (35.7%)	2181 (54.9%)
	Black	318 (8.0%)	451 (11.3%)	769 (19.3%)
	Asian	17 (0.4%)	61 (1.5%)	78 (2.0%)
	Other	330 (8.3%)	617 (15.5%)	947 (23.8%)
**Ethnicity**	Non-Hispanic	1166 (29.3%)	2020 (50.8%)	3186 (80.2%)
	Hispanic	213 (5.4%)	447 (11.2%)	660 (16.6%)
	Other	47 (1.2%)	82 (2.1%)	129 (3.2%)

**Education**	No high school	549 (13.8%)	970 (24.4%)	1519 (38.2%)
	High school	385 (9.7%)	621 (15.6%)	1006 (25.3%)
	College+	492 (12.4%)	958 (24.1%)	1450 (36.5%)
**Employment**	Employed	356 (9.0%)	843 (21.2%)	1199 (30.2%)
**Income**	<25k	394 (9.9%)	635 (16.0%)	1029 (25.9%)
	25k–50k	241 (6.1%)	460 (11.6%)	701 (17.6%)
	50k–100k	306 (7.7%)	595 (15.0%)	901 (22.7%)
	100k–200k	171 (4.3%)	323 (8.1%)	494 (12.4%)
	>200k	314 (7.9%)	536 (13.5%)	850 (21.4%)
**Insurance**	Insured	1341 (33.7%)	2415 (60.7%)	3756 (94.5%)
**Marital Status**	Married	656 (16.5%)	1155 (29.1%)	1811 (45.6%)
**Medications**	Sulfonylureas	980 (24.7%)	1071 (27.0%)	2051 (51.6%)
	Insulin	1272 (32.0%)	1867 (47.0%)	3139 (79.0%)
	SGLT2i	783 (19.7%)	1143 (28.8%)	1926 (48.5%)
	Metformin	1321 (33.2%)	2209 (55.6%)	3530 (88.8%)
	DPP4i	1126 (28.3%)	1480 (37.2%)	2606 (65.6%)
	Statin	1364 (34.3%)	2212 (55.6%)	3576 (90.0%)
**Lifestyle**	Alcohol	821 (20.7%)	1706 (42.9%)	2527 (63.6%)
	Smoking	101 (2.5%)	179 (4.5%)	280 (7.0%)
**GLP-1 RA Type**	Semaglutide	318 (8.0%)	910 (22.9%)	1228 (30.9%)
	Dulaglutide	447 (11.2%)	899 (22.6%)	1346 (33.9%)
	Exenatide	200 (5.0%)	147 (3.7%)	347 (8.7%)
	Liraglutide	438 (11.0%)	572 (14.4%)	1010 (25.4%)
	Other	23 (0.6%)	21 (0.5%)	44 (1.1%)
**Comorbidities**	CKD	647 (16.3%)	737 (18.5%)	1384 (34.8%)
	Heart failure	475 (12.0%)	526 (13.2%)	1001 (25.2%)
**Other Medications**	Thiazide	1289 (32.4%)	2117 (53.3%)	3406 (85.7%)
	Beta-blocker	1316 (33.1%)	2123 (53.4%)	3439 (86.5%)

	Steroid	1308 (32.9%)	2137 (53.8%)	3445 (86.7%)
**Continuous Variables (Mean ± SD)**	Age (years)	59.99 ± 11.20	58.35 ± 12.03	58.93 ± 11.76
	BMI	35.92 ± 11.01	35.80 ± 8.21	35.84 ± 9.31
	Duration of diabetes (years)	13.99 ± 5.10	9.85 ± 4.52	11.34 ± 5.14
	Baseline HbA1c (%)	8.65 ± 1.70	8.18 ± 1.92	8.35 ± 1.87
	SBP	130.17 ± 11.07	129.68 ± 11.00	129.86 ± 11.03
	DBP	75.61 ± 6.87	76.92 ± 7.32	76.45 ± 7.19
	LDL	89.49 ± 25.82	89.47 ± 28.35	89.48 ± 27.47
	HDL	44.03 ± 11.07	44.88 ± 11.59	44.57 ± 11.41
	Total cholesterol	169.92 ± 31.51	169.33 ± 35.63	169.54 ± 34.20
	Sodium	137.76 ± 5.63	138.17 ± 5.31	138.02 ± 5.43
	Chloride	102.21 ± 3.42	102.37 ± 4.44	102.31 ± 4.11
	Calcium	9.35 ± 2.22	9.38 ± 2.05	9.37 ± 2.11
	Magnesium	2.39 ± 5.30	2.43 ± 4.61	2.41 ± 4.87

Table S2 represents how participants were classified according to glycemic control status at follow-up, defined as achieving HbA1c <7% after GLP-1 receptor agonist initiation. Categorical variables are presented as frequencies and percentages, and continuous variables are presented as mean ± standard deviation (SD). Percentages are calculated based on the total study population (N = 3,975).

Table S3: Sensitivity Analysis of Machine Learning Models for Predicting Clinically Meaningful Weight Loss Following GLP-1 Receptor Agonist Therapy

Model	AUC	Accuracy	Precision	Sensitivity	F1-score
LR	0.61 ± 0.02	0.58 ± 0.001	0.48 ± 0.001	1.000 ± 0.00	0.65 ± 0.00
RF	0.72 ± 0.01	0.66 ± 0.01	0.62 ± 0.01	0.74 ± 0.02	0.68 ± 0.01
XGBoost	0.72 ± 0.01	0.66 ± 0.02	0.62 ± 0.02	0.79 ± 0.02	0.69 ± 0.02
SVM	0.61 ± 0.01	0.57±0.01	0.55±0.01	0.62±0.01	0.58±0.01

NN	0.61 ± 0.01	0.55 ± 0.01	0.53 ± 0.01	0.54 ± 0.02	0.536 ± 0.01
LightGBM	0.72 ± 0.01	0.66 ± 0.01	0.61 ± 0.01	0.81 ± 0.02	0.70 ± 0.01
Catboost	0.72 ± 0.01	0.66 ± 0.02	0.61 ± 0.01	0.81 ± 0.02	0.69 ± 0.01

Table S3 represents performance of machine learning models for predicting clinically meaningful weight loss, defined as ≥5% reduction from baseline body weight among GLP-1 receptor agonist users. Results are presented as mean ± standard deviation from 10-fold cross-validation. Performance metrics include accuracy, precision, sensitivity (recall), and F1-score. This sensitivity analysis was conducted to evaluate whether model performance remained robust when a clinically meaningful weight-loss threshold was used instead of the primary BMI-based outcome definition. Tree-based ensemble models maintained the highest performance under the clinically meaningful ≥5% weight-loss definition, indicating robustness of the primary findings to alternative outcome definitions. Model performance decreased modestly after removal of baseline BMI; however, several models maintained acceptable discrimination and classification performance, suggesting that prediction was not solely driven by baseline BMI.

Table S4. Sensitivity Analysis of Machine Learning Models for Weight Loss Prediction After Excluding Baseline BMI from the Predictor Set

**Model**	**AUC**	**Accuracy**	**Precision**	**Sensitivity**	**F1-score**
LR	0.90 ± 0.01	0.792 ± 0.012	0.506 ± 0.017	0.881 ± 0.016	0.642 ± 0.016
RF	0.90± 0.01	0.859 ± 0.009	0.744 ± 0.034	0.511 ± 0.034	0.605 ± 0.029
XGBoost	0.90 ± 0.01	0.863 ± 0.009	0.711 ± 0.031	0.592 ± 0.020	0.646 ± 0.020
SVM	0.65 ± 0.01	0.620 ± 0.009	0.298 ± 0.010	0.587 ± 0.030	0.395 ± 0.015
NN	0.61 ± 0.02	0.711 ± 0.015	0.304 ± 0.028	0.280 ± 0.038	0.290 ± 0.028
LightGBM	0.90 ± 0.01	0.838 ± 0.008	0.587 ± 0.016	0.790 ± 0.022	0.673 ± 0.013
CatBoost	0.90 ± 0.01	0.840 ± 0.010	0.593 ± 0.020	0.775 ± 0.026	0.672 ± 0.019

Table S4 indicates performance of machine learning models for predicting achievement of the primary weight loss outcome (BMI <30 kg/m² at follow-up) following GLP-1 receptor agonist therapy after removal of baseline body mass index (BMI) from the predictor set. Results are presented as mean ± standard deviation from 10-fold cross-validation. Performance metrics include accuracy, precision, sensitivity (recall), and F1-score. This sensitivity analysis was

conducted to assess the extent to which model performance depended on baseline BMI and to evaluate the contribution of other demographic, clinical, laboratory, medication-related, and socioeconomic predictors to weight outcome prediction.

Table S5. Sensitivity Analysis of Machine Learning Models for Glycemic Control Prediction After Excluding Baseline HbA1C from the Predictor Set

**Model**	**AUC**	**Accuracy**	**Precision**	**Sensitivity**	**F1-score**
LR	0.77 ± 0.01	0.664 ± 0.011	0.659 ± 0.006	0.987 ± 0.009	0.790 ± 0.006
RF	0.78±0.01	0.730 ± 0.017	0.748 ± 0.017	0.874 ± 0.015	0.806 ± 0.011
XGBoost	0.79 ± 0.01	0.726 ± 0.018	0.766 ± 0.019	0.826 ± 0.012	0.795 ± 0.011
SVM	0.77 ± 0.02	0.704 ± 0.015	0.809 ± 0.020	0.705 ± 0.017	0.753 ± 0.012
NN	0.69 ± 0.03	0.669 ± 0.022	0.737 ± 0.021	0.753 ± 0.017	0.745 ± 0.016
LightGBM	0.77 ± 0.01	0.713 ± 0.014	0.788 ± 0.016	0.756 ± 0.015	0.772 ± 0.010
CatBoost	0.78 ± 0.01	0.717 ± 0.015	0.790 ± 0.021	0.762 ± 0.012	0.775 ± 0.010

Table S5 shows performance of machine learning models for predicting glycemic control following GLP-1 receptor agonist therapy after removal of baseline hemoglobin A1c (HbA1c) from the predictor set. Results are presented as mean ± standard deviation from 10-fold cross-validation. Performance metrics include area under the receiver operating characteristic curve (AUC), accuracy, precision, sensitivity (recall), and F1-score. This sensitivity analysis was conducted to evaluate the extent to which model performance depended on baseline HbA1c and to assess the contribution of other demographic, clinical, laboratory, medication-related, and socioeconomic predictors to glycemic control prediction. Although model performance declined following removal of baseline HbA1c, Random Forest and XGBoost continued to demonstrate acceptable performance, indicating that additional demographic, clinical, and treatment-related predictors contributed substantially to glycemic control prediction.

Abbreviations: AUC, area under the receiver operating characteristic curve; BMI, body mass index; CatBoost, categorical boosting; DPP4i, dipeptidyl peptidase-4 inhibitor; GLP-1 RA, glucagon-like peptide-1 receptor agonist; HbA1c, hemoglobin A1c; LightGBM, light gradient boosting machine; LR, logistic regression; NN, neural network; RF, random forest; SGLT2i,

sodium-glucose cotransporter-2 inhibitor; SVM, support vector machine; XGBoost, extreme gradient boosting.
